# New graduate nurses’ experiences in a clinical specialty: a follow up study of newcomer perceptions of transitional support

**DOI:** 10.1186/s12912-017-0236-0

**Published:** 2017-07-28

**Authors:** Rafic Hussein, Bronwyn Everett, Lucie M. Ramjan, Wendy Hu, Yenna Salamonson

**Affiliations:** 1 Western Sydney University, School of Nursing and Midwifery, Locked Bag 1797, Penrith, NSW 2751 Australia; 2 Centre for Applied Nursing Research (CANR), Ingham Institute for Applied Medical Research, Liverpool, NSW 2170 Australia

**Keywords:** Nurses, Newcomer nurses, New graduate nurse, Newly qualified nurses, Newly licensed nurses, Professional support, Nurse practice environment, Clinical supervision, Transition, Retention, Mixed methods

## Abstract

**Background:**

Given the increasing complexity of acute care settings, high patient acuity and demanding workloads, new graduate nurses continue to require greater levels of support to manage rising patient clinical care needs. Little is known about how change in new graduate nurses’ satisfaction with clinical supervision and the practice environment impacts on their transitioning experience and expectations during first year of practice. This study aimed to examine change in new graduate nurses’ perceptions over the 12-month Transitional Support Program, and identify how organizational factors and elements of clinical supervision influenced their experiences.

**Methods:**

Using a convergent mixed methods design, a prospective survey with open-ended questions was administered to new graduate nurses’ working in a tertiary level teaching hospital in Sydney, Australia. Nurses were surveyed at baseline (8–10 weeks) and follow-up (10–12 months) between May 2012 and August 2013. Two standardised instruments: the Manchester Clinical Supervision Scale (MCSS-26) and the Practice Environment Scale Australia (PES-AUS) were used. In addition to socio-demographic data, single –item measures were used to rate new graduate nurses’ confidence, clinical capability and support received. Participants were also able to provide open-ended comments explaining their responses. Free-text responses to the open-ended questions were initially reviewed for emergent themes, then coded as either positive or negative aspects of these preliminary themes. Descriptive and inferential statistics were used to analyse the quantitative data and the qualitative data was analysed using conventional content analysis (CCA). The study was approved by the relevant Human Research Ethics Committees.

**Results:**

Eighty seven new graduate nurses completed the follow-up surveys, representing a 76% response rate. The median age was 23 years (Range: 20 to 53). No change was seen in new graduate nurses’ satisfaction with clinical supervision (mean MCSS-26 scores: 73.2 versus 72.2, *p* = 0.503), satisfaction with the clinical practice environment (mean PES-AUS scores: 112.4 versus 110.7, *p* = 0.298), overall satisfaction with the transitional support program (mean: 7.6 versus 7.8, *p* = 0.337), satisfaction with the number of study days received, orientation days received (mean: 6.4 versus 6.6, *p* = 0.541), unit orientation (mean: 4.4 versus 4.8, *p* = 0.081), confidence levels (mean: 3.6 versus 3.5, *p* = 0.933) and not practising beyond personal clinical capability (mean: 3.9 versus 4.0, *p* = 0.629).

Negative responses to the open-ended questions were associated with increasing workload, mismatch in the level of support against clinical demands and expectations. Emergent themes from qualitative data included i) orientation and Transitional Support Program as a foundation for success; and ii) developing clinical competence.

**Conclusions:**

While transitional support programs are helpful in supporting new graduate nurses in their first year of practice, there are unmet needs for clinical, social and emotional support. Understanding new graduate nurses’ experiences and their unmet needs during their first year of practice will enable nurse managers, educators and nurses to better support new graduate nurses’ and promote confidence and competence to practice within their scope.

## Background

Acute healthcare settings in Australia and developed countries are rapidly evolving [1] and becoming increasingly complex [2]. For newly graduated registered nurses (NGNs), transitioning from university to practice in acute settings remains challenging, stressful and emotionally exhausting [3] as they strive to deliver safe nursing care amidst heavy workloads, increased accountability and responsibility for their patient care [4]. Concerns about new graduate nurses’ ability to cope and deliver safe nursing care have contributed to the development of transitional support programs alongside various forms of clinical supervision to promote the development of clinical proficiency, support professional development and improve new graduate nurse retention [5].

To fully comprehend the transitional experience of new graduates it is important to understand their clinical environment and workplace conditions. New graduate nurses continue to enter a work environment characterised by nursing staff shortages, increasing patient acuity [6] and at times limited access to clinical support [7]. Although a positive workplace environment facilitates more effective transition of graduate nurses and significantly influences their job satisfaction [6], negative experiences have been found to result in feelings of heightened work stress for up to one year after graduation, with contributory factors including poor work environments, poor clinical supervisors and poor nurse-doctor relations [8]. Not only do these early experiences impact on new graduate nurses’ levels of satisfaction but they can influence long term career intentions [5]. Of concern is that current research on the experiences of first year nurses still reflects the findings of the research on their counterparts a decade earlier; that is, they still struggle to meet expectations placed on them, face difficulties to manage unreasonable workloads, high levels of stress, burnout and feeling at times unsafe [9].

New graduate nurses’ experiences in the first year of practice are often described as overwhelming and stressful as they strive to apply newly acquired skills, deliver quality patient care and ‘fit in’ [10]. Importantly, the first year of practice is also a time of high attrition with rates of up to 27% reported in the literature [11]. These concerns have contributed internationally to the development of new graduate programs [12], often referred to as transitional programs, to promote clinical proficiency, support NGNs’ professional development and improve retention.

Numerous studies have reported the impact of workplace stress, uncivil behavior and burnout on the retention of new graduate nurses [9, 11, 13]. However, how best to facilitate newcomer transition in acute care settings remains a subject of ongoing research. Although there is consensus in the literature that a supportive organizational environment (both at the ward and organizational level) is needed for the safe and successful integration of novice nurses, few authors have detailed the experiences and perceptions of new graduate nurses and how these change over time. Bauer, Bodner, Erdogan, Truxillo and Tucker [14] describe the process of newcomer nurses being socialized into organizations, sometimes referred to as ‘onboarding’, during which time they acquire the knowledge, attitudes and behaviors to perform effectively and adjust to their work surroundings [15]. However, this process has been shown to be negatively impacted by rising patient acuity and understaffing and in some cases, exacerbated by fear of failure [16].

Transitional programs are one intervention to address these challenges; the format varies, but they are designed to assist new graduate nurses’ transition into the nursing workforce and profession within an institutional context. Over 12 months, the program offers new graduates exposure to a variety of clinical settings including facility and ward orientation, 2–3 ward rotations, 4–5 pre-planned study days, formal and informal clinical supervision. Such programs aim to assist novice nurses with facility and ward orientation to consolidate theoretical learning, critical skills and judgement in their new professional role [11]. To ensure a successful transition from a novice nurse to competent registered nurse (RN), it is argued that structured professional development programs are provided [17] in a supportive manner to help newly graduated nurses integrate into organizational systems and processes [7].

Given the implications for nursing workforce retention, it is thus important to examine the effectiveness of transitional support programs and the factors associated with positive and negative new graduate experiences. The overall aim of this study was to examine change in new graduate nurses’ perceptions over a 12-month transitional support program (TSP), also commonly known as nurse residency program. Specifically, this study sought to (i) identify elements of clinical supervision that influenced new graduates’ experiences during the program; (ii) to examine changes in new graduate nurses’ perceptions and clinical supervision, confidence levels, satisfaction with the orientation program and their practice environment over the 12-month transitional period and (iii) explore their experiences during the transitional period and identify change between baseline and follow-up.

## Methods

**Fig. 1:**
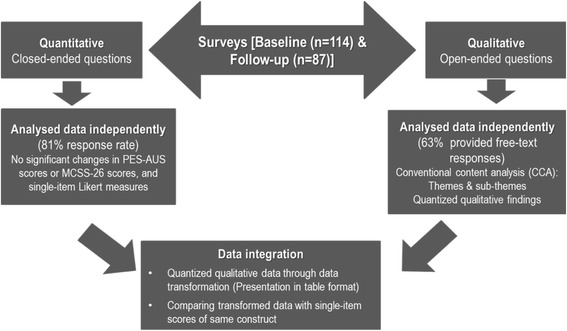
Overview of Convergent Mixed Methods Design [3, 19]

This convergent mixed methods study was part of a larger project [18] which evaluated the effectiveness of clinical supervision practice for new graduate nurses in an acute care setting (Fig. 1). The value of mixed methods research can be dramatically enhanced through the integration of quantitative and qualitative data [3, 19].

In this paper, we integrate an analysis of quantitative data with a separate analysis of the qualitative open-ended responses from a survey completed by new graduate nurses enrolled in a transitional support program. The survey was initially administered 8–10 weeks after the commencement of the transitional support program and again to the same participants 10–12 months after commencement. These data collection points were selected for pragmatic reasons; that is they coincided with programmed study days and ensured participants had sufficient time in each of their rotations to report their transitioning experience and clinical competence.

### Study setting and participants

The study was conducted in a principal referral and teaching hospital in Sydney, Australia. The hospital has 855 beds and employs over 1500 nursing staff across a range of clinical streams and specialities, including state-wide services in critical care and trauma. All new graduate nurses (*n =* 140) enrolled in the 12-month transitional support program were invited to participate in this study at facility orientation, study days and by emailed invitations. Programmed study days are pre-allocated study days for new graduates throughout the transitional support programme. ‘Orientation days’ refers to the number of days new graduate nurses were given as supernumerary on the ward. This means they were ‘buddied up’ for the shift or preceptored by another registered nurse or clinical nurse educator. Data collection for this study was conducted between May 2012 and August 2013 during group TSP study days, shift overlap times on the ward and other times convenient to participants. Participants completed the self-report instruments independently.

### Data collection

Participant characteristics assessed in this study included i) age; ii) gender; iii) history of previous paid employment, and iv) previous nursing experience. Single-item Likert scale measures were used to assess how frequently (0 = never, 10 = always) NGNs were placed in clinical situations where they did not feel confident or which were beyond their clinical capability. In addition, three practice environment factors were included: i) assigned unit (critical care or non-critical care area); ii) level of satisfaction with unit-based orientation using a single-item question and; iii) satisfaction with the clinical supervision offered within the TSP. Participants were asked to give a reason for their rating or elaborate by providing an example for the single-item measures. Also administered at baseline and follow up were two validated instruments, the 26-item Manchester Clinical Supervision Scale (MCSS-26) used to assess NGN perception of the quality of clinical supervision [20], and the Practice Environment Scale - Australia (PES-AUS) used to assess satisfaction with their clinical practice environment [21]. The PES-AUS was modified with a midpoint of 3 = ‘unsure’, as it was anticipated that some new graduates may not be familiar with some items of the scale. In this study, Cronbach’s alpha for both instruments were 0.90 and 0.91 respectively, indicating high internal consistency.

Ethics approval was granted by Western Sydney University and South Western Sydney Local Health District Human Research Ethics Committees (H10055, LNR/11/LPOOL/510). Written informed consent was obtained from all participants. Permission to use the MCSS-26 and PES-AUS was obtained by the authors.

### Data analysis

Quantitative data were analyzed using the statistical software package, IBM SPSS Statistics Version 22 [4]. Continuous variables were assessed for normality using the Kolmogorov-Smirnov test, and expressed as median and range. Categorical variables were summarized as frequencies and percentages. To examine for change in the study cohorts’ responses between baseline and follow-up, we used Pearson’s chi-square for categorical variables, paired *t*-test or Wilcoxon signed rank test for changes between baseline and follow-up. A *p*-value of <0.05 was considered as statistically significant.

A Conventional Content Analysis (CCA) technique was used to analyze the open-ended responses. This approach allows the analysis (themes and names for the themes) to be derived from the open-ended responses, rather than being preconceived [22]. The open-ended responses were read multiple times by the first author (RH) to achieve immersion [22]. Responses were then read for frequently repeated words (e.g. support, workload, skills), denoting main views on experiences and transition were highlighted within an excel spreadsheet. First impressions of open-ended responses were noted and formed the basis of development of categories or ‘sub-themes’ for grouping under main ‘themes’.

Open-ended responses to the single item measures were reviewed for emergent themes by two researchers (RH and LR). Initially 20% of the free text responses were coded independently by two researchers (RH & YS), categorizing the text as either positive or negative aspects of these preliminary themes. Any differences in coding were then discussed to achieve consensus before the continuation of further text coding. RH completed the remainder of the coding. Data integration was achieved by transforming (‘quantitizing’) the qualitative data into numerical form [19].

Using the six subthemes as categories, the frequencies of the free-text responses were grouped into positive and negative dimensions, based on the number of times a code referring to a sub-theme was found in a participants’ survey. A numerical value of “1” was given for each positive comment and a score of “0” if the comment was negative. An aggregate score for each subtheme was thus computed (Table 1). The qualitative responses were then ‘transformed’ into quantitative data, then integrated with illustrative examples from the original dataset [23].

**Table 1:** Positive and negative comments of new graduate nurses at baseline and follow-up

Theme	No.	Category	Frequency of comments					
			Baseline			Follow-up		
			Positive	Negative	Balance	Positive	Negative	Balance
1. Orientation and Transitional Support Program as foundation for success	1.1	Instrumental support during transition	33 (43%)	36 (22%)	−3	22 (51%)	31 (30%)	−9
	1.2	Understanding the clinical capabilities of the new graduate	14 (18%)	31 (19%)	−17	4 (9%)	9 (8%)	−5
	1.3	Becoming one of the team or part of the team	3 (4%)	3 (2%)	0	2 (5%)	2 (2%)	0
2. Developing clinical competence	2.1	Appropriate workload and working within scope of practice	8 (10%)	47 (28%)	−39	2 (5%)	36 (34%)	−34
	2.2	Adequate skill mix	9 (12%)	11 (6%)	−2	5 (12%)	5 (5%)	0
	2.3	Building clinical confidence and competence	10 (13%)	39 (23%)	−29	8 (18%)	22 (21%)	−14
		Subtotal	77 (100%)	167 (100%)	−90	43 (100%)	105 (100%)	−62
			Baseline Balance			Follow-up Balance		

## Results

### Quantitative findings

#### Sample characteristics

A total of 140 new graduate nurses enrolled in the transitional program. One hundred and fourteen new graduates (81%) completed the baseline survey, and of these, 87 (76%) completed follow-up surveys. There were no statistically significant differences in age, MCSS-26 or PES-AUS scores between responders and non-follow-up responders.

The median age was 23 years (Range: 20 to 53) and over three-quarters of the sample were female (78%). Over half of the new graduates had previous nursing experience, with most previously employed as Assistants in Nursing (AINs) (unlicensed workers). During the two clinical rotations within the transitional support program, approximately two-thirds (63%) of new graduates worked in non-critical care areas with the remainder (37%) allocated to work in critical care areas.

#### Experiences over time: Baseline vs follow up

There was no change in the new graduate nurses’ satisfaction with clinical supervision over the two time periods (mean MCSS-26 scores: 73.2 versus 72.2, *p* = 0.503). Similarly, there were no significant differences in: i) satisfaction with the clinical practice environment (mean PES-AUS scores: 112.4 versus 110.7, *p* = 0.298); ii) the overall satisfaction with the transitional support program (mean: 7.6 versus 7.8, *p* = 0.337); iii) satisfaction with the number of study days received (mean: 4.4 versus 4.7, *p* = 0.72); iv) orientation days received (mean: 6.4 versus 6.6, *p* = 0.541); v) unit orientation (mean: 4.4 versus 4.8, *p* = 0.081); vi) confidence levels (mean: 3.6 versus 3.5, *p* = 0.933) and vii) not practising beyond personal clinical capability (mean: 3.9 versus 4.0, *p* = 0.629), over the two time periods.

### Qualitative findings

Almost two-thirds *n* = 72 (63%) of participants provided free text responses to either the open-ended questions at baseline and/or follow-up. Two themes, each with three subthemes encapsulated their experiences during this period. These are listed in Table 1, and presented below with illustrative quotes.

#### Theme 1: Orientation and TSP as a foundation for success

The majority of participants who responded commented that the transitional support program and orientation was essential for successful transition. Those who indicated they received good supernumerary support and orientation felt they were *"formally introduced to the team which was satisfying and welcoming for new staff".*

*Overall the TSP team have provided a great deal of clinical and emotional support throughout the year. The program has been useful in transitioning into the hospital setting (Participant 2.35).*

### Sub-theme 1: Instrumental support during transition

In addition to a comprehensive orientation program to ward or unit, continuing support from clinical nurse educators (CNEs), nurse unit managers (NUMs), clinical nurse specialists and registered nurses was identified as crucial during this period as it fostered acceptance and learning.

*The support was exceptional. CNE was very thorough and supportive alongside the NUM (Participant 3.6).*

*Good experiences, worked in a supportive environment, also got continual support after formal orientation (Participant 4.22).*

### Sub-theme 2: Understanding the clinical capabilities of the new graduate

Some ward staff though had unrealistic expectations of the clinical capabilities of the new graduates, and others spent minimal time orientating them to the ward which was unhelpful; as newcomers they required sufficient time to familiarise with ward, layout, equipment and policies.

*I found that I didn’t have very much time to get orientated and was pushed into the deep end (Participant 3.9).*

*Three Cardiothoracic patients at one given time, 1 pt. on inotropes, noradrenaline and dobutamine. Another post coronary artery bypass graft [CABG], another tracheostomy. Plus there was a met call that day (participant 2.32).*

### Sub-theme 3: Becoming one of the team

Surprisingly, new graduates were not always formally introduced to other staff members in the ward or unit. One participant suggested the following:

*Have a day to introduce me to ward (staff) (Participant 4.17).*

Despite this, being an integral member of the team was also evident, as illustrated by this comment:

*The ward staff have been very supportive and often give tips/input on what can be done on a particular situation. (Participant 2.9).*

#### Theme 2: Developing clinical competence

Many of the NGNs felt that the transitional support program provided them with opportunities to develop their clinical competence. However, access to opportunities for further development and support varied, depending on the availability and expertise of the TSP coordinators, the after-hours nurse educator, the ward-based clinical nurse educators and clinical nurse specialists, team leaders and other senior staff. The variability in opportunities influenced the capacity of novice nurses to develop their clinical competence, which was further compounded by increasing workloads, and nursing skill mix. Being confronted by unexpected clinical situations such as the Medical Emergency Team (MET) calls, deteriorating patients, dealing with aggressive patients and challenging families were some of the examples cited by participants as illustrated by the comment below:

*I’ve found that I have been put in situations I have had little exposure to with minimal help at hand. Although some senior staff may help, it may take some convincing. (Participant 3.9).*

Nevertheless, other novice nurses were able to continue to develop their clinical competence, albeit with some difficulty:

*Two months was enough for me to positively develop skills and knowledge. However, emotionally it was very hard and draining (Participant 4.15).*

### Sub-theme 1: Appropriate workload and working within scope of practice

Not surprisingly, some new graduates felt that the expectations and workload were unrealistic due to high patient acuity and staff shortages. They lamented that at times, they were working outside their scope of practice with heavy patient loads.

*Ten patients, multiple admissions and discharges, time consuming procedures* e.g. *dressings, blood transfusions and post-op patients with only an undergraduate nurse (Participant 3.41).*

*All 5 patients were total nursing care. Had never seen or used the drains, dressings or TPN. No help or not much help available. Ward assumes I know and seem cranky if I say I don’t know… (Participant 3.32).*

### Sub-theme 2: Inadequate skill mix

The participants also mentioned that they were often expected to deliver quality care despite ineffective skill mix. This was further complicated by high patient acuity, MET calls and rapid turnover of patients. At times new graduate nurses were paired with assistants in nursing, having little capacity to support their work partner, while at the same time, working beyond their own capabilities. To illustrate this, one new graduate highlighted:

*I felt as though it was often new graduate nurses on the ward were allocated unfairly by senior staff members ... on numerous occasions new graduates were allocated to the heaviest teams with the casual AINs ...while senior staff allocated themselves with ENs and RNs (Participant 2.35).*

### Sub-theme 3: Building clinical confidence and competence

Despite the many difficulties, most new graduates had sufficient confidence in handling emergent situations because of the adjunct support around them. One participant remarked:

*Had patients needing MET calls for various conditions (VT, VF, desaturation). I did not feel confident taking care of these patients on my own but had help around me at these times (Participant 4.17).*

Nevertheless, others at times lacked confidence and struggled to manage patients:

*I felt insecure.... and I was not sure what to do, such as when discharging a patient and what to do when facing an emergency situation although I have been told what to do (Participant 3.16).*

*A patient (HDU) clinically deteriorated and felt very uncomfortable, useless, dumb as I did not know what to do and the team took over (Participant 4.15).*

#### Quantized qualitative findings

There were numerous comments about ‘Instrumental Support during Transition’ [Subtheme 1.1] at both baseline (Total: 69) and follow-up (Total: 53) with most comments about this category being more negative at follow-up. In the second subtheme ‘Understanding the Clinical Capabilities of the New Graduate’ [Subtheme 1.2], most comments at baseline and follow-up were negative, however, the proportion of these responses at follow-up were less (9) compared to baseline (31). A few comments were made about, ‘Becoming one of the team [subtheme 1.3]; at both at baseline and follow-up.

In the second theme, most comments from new graduate nurses about ‘Appropriate workload and working within scope of practice’ [subtheme 2.1.] were overwhelmingly negative. At baseline 47 of 55 responses about workload were negative (85%) and at follow-up 36 of 38 were also negative (95%). In relation to ‘Adequate skill mix’ [subtheme 2.2], new graduates made fewer comments and the overall frequencies of positive and negative responses at both baseline and follow-up were equal. The majority of new graduates commented on their feelings of a lack of ‘Clinical confidence and competence’ at baseline [subtheme 2.3], but overall there were less negative comments about this at follow-up. Overall, the free text responses to the open-ended questions at follow-up were less negative (Table 1).

### Integrated findings

Although no statistically significant changes in new graduate nurses’ satisfaction with clinical supervision, orientation days received, overall experience with the transitional support program, and the clinical practice environment were detected in the quantitative data, a count of the frequency of coded comments suggested new graduates were in fact more satisfied at follow-up. For example, when new graduates were asked about their satisfaction with their orientation and transitional support program, the number of negative responses provided at follow-up (42) was less than at baseline (70). Similarly, the number of negative comments related to the sub-theme, ‘building clinical confidence and competence’ decreased from 39 at baseline to 22 at follow-up while the number of negative comments related to the sub-theme ‘Appropriate workload and working within scope of practice’, decreased from 47 at baseline to 36 at follow-up (Table 1).

## Discussion

This study sought to examine new graduate nurses’ perceptions and experiences of clinical supervision during a 12-month transitional support program and the changes experienced over this period.

Although it was anticipated that new graduate nurses’ satisfaction with their transitional support program would increase over time, this was not reflected in their MCSS (clinical supervision) or PES-AUS (practice environment) scores which were similar at baseline and follow-up. This could reflect the timing of the initial data collection which occurred 8–10 weeks after participants commenced their TSP. It may be that the most difficult transition period had already passed given participants had already completed a minimum of 2 months of their first rotation. Duchscher [24] identified the most intense adjustments occurred during the first 1–4 months of the TSP which coincides with the initial data collection. Thus, it may be that ‘the worst of it was over’ and participants’ initial clinical supervision and practice environment scores reflected this.

However, new graduates did make less negative responses to the open-ended questions at follow-up than baseline, suggesting that overall new graduate nurses were more satisfied at the completion of their TSP. It is not clear why an increase in satisfaction was not identified using the PES-AUS and MCSS but it could be that while validated in mixed groups of nurses, the instruments were not sensitive enough to detect change over time in a smaller group of newly graduated nurses. It might also be that negative experiences stemming from lack of support, transition shock [25], practice readiness [26], lack of confidence in clinical practice [5, 18, 27] and at times the high levels of stress experienced in the acute care setting impacted on the low follow-up scores. Interestingly, [28] reported a V-shaped pattern in new graduates’ satisfaction scores in a residency program; that is, a decline from baseline to 6-months, then returning to baseline scores at 12-months suggesting that new graduates adjust after the initial period of the support program. In the current study, it may be that the transition to a different clinical specialty after the first rotation resulted in new graduates once again feeling out of their depth, which was reflected in their satisfaction scores.

Overall, new graduate nurses were satisfied with the number of study days they received throughout the program however, it was concerning that they reported low satisfaction scores for unit orientation at both baseline (reflecting their first rotation) and follow-up (reflecting their second rotation). While new graduates were generally satisfied with orientation to specialty areas such as the intensive care unit (ICU), paediatric intensive care (PICU) and coronary care unit (CCU), Recovery/Anaesthetics or areas such as aged care where orientation was provided over a longer period of time, many new graduates felt that the time allocated to orientation was insufficient. In particular, additional time to become familiar with ward routines, layout, equipment and policies was needed. It is possible that staff expectations of new graduate nurses’ readiness to take on a patient load were higher in the knowledge that this was a second rotation. This is consistent with studies where clinicians have questioned the need for transition programs, arguing that new graduates should be ‘practice-ready’ [29] for the workplace [27].

These findings are reflected in the qualitative data with nurses commenting that they were “thrown in the deep end” (Participant 3.6) and “put in a room and told to read policies” (Participant 3.45). It is unclear if the low satisfaction scores with ward orientation were the result of inadequate support from the ward educators or educators working different schedules to the new graduates. Other factors such as staff shortages, a mismatch between patient acuity and skill mix could also possibly explain the low satisfaction scores. These findings have important implications for developing well-structured ward based orientation programmes to support new graduate nurses’ needs.

Interestingly, those participants who reported receiving satisfactory supernumerary support and orientation also reported they felt welcomed and part of the team and were able to “find their feet”. This highlights the need for ward staff to understand the clinical capabilities of new graduate nurses and not to have unrealistic expectations of them.

Two single-item measures asked graduate nurses how often they had been placed in a clinical situation where they felt the clinical workload was beyond their level of clinical capability, and where they did not feel confident about handling the clinical situation. While it was reassuring to find overall low scores indicating these situations occurred rarely, the qualitative findings suggested staffing ratios, adequate skill mix, patient acuity, lack of instrumental support and clinical workload were common reasons for new graduates being placed in a situation which they felt was beyond their clinical capability. This is consistent with previous research on new graduate nurses experiences during transition which highlighted the inconsistency between support given and required [9].

It also appeared some new graduates were allocated patients or required to perform skills that were beyond their scope of practice as reflected by the large number of negative responses related to appropriate workload and working within scope of practice [Subtheme 2.1]. For many new graduates in this study, the workload seemed to be stressful and beyond their abilities, particularly on transition from areas which had a reduced patient load, such as ICU, to an area that required them to care for 8–10 patients. This finding suggests senior staff who allocate workloads to NGNs need to consider the NGN’s previous rotation and take this into account when allocating patient loads or requesting them to perform skills they may not yet have encountered. The assumption that nurses on their second rotation have developed proficiency in time management and managing increased patient loads may not be true for graduates who have been working in specialty areas such as emergency departments or intensive care units. In an integrative review to identify best practices of formal new graduate transition to practice programs, Rush et al. [27] identified the need for formal support in the 6–9 month period. Similarly, Duchscher’s [25] work on female graduates from a 4-year Canadian baccalaureate program also identified the 5–7 month time point as one where new graduates experienced a ‘crisis of confidence’. In the current study, this time point coincides with the beginning of the new graduates’ second rotation and may help explain the high number of negative comments about workload and working within scope of practice.

Encouragingly, in this study participants reported they were rarely placed in situations where they did not feel confident about handling clinical situations and if this did occur, there was good support from seniors who helped them to manage the situation. Situations which were likely to cause new graduates to feel they were not confident most commonly related to workload and skill-mix issues [Subtheme 2.1].

Consistent with previous findings new graduate nurses volunteered that they were occasionally placed in stressful situations [30]. Deflated by the lack of clinical support, some new graduates felt they were being “set up to fail” (Participant 4.8). Scott et al. [5] highlight that critical to providing a supportive work environment for new graduate nurses is the need to ensure that initial orientation to the clinical environment is accompanied with an affirmative experience into the organization. This may include an introduction to managers and co-workers, outlining and building graduate nurses’ understanding of organizational policy, process, mission, vision, values, and making clear to NGNs their role in achieving quality care and patient safety [6]. The early dissemination of workplace resources to new graduate nurses such as access to information, necessary resources to getting the job performed and opportunity for growth and development are essential for their empowerment [30]. Laschinger et al. [13] found that new graduate nurses who felt empowered in the work environment and supported to accomplish their workload reduced the likelihood of burnout. Overall, findings from this study support contemporary evidence from the United States suggesting that nurse residency programs assist newly licensed nurses in facing the challenges in applying recently learnt knowledge and skills in acute care settings [31].

### Limitations

This study was undertaken at a large, tertiary referral hospital which provides services for more than 800,000 residents in the District, including many with high levels of illness severity and therefore the findings may not be generalizable to other study settings. Although the MCSS and PES-AUS are standardised measures, and have been shown to be reliable, the PES-AUS was modified for this study from a 4-point Likert scale to a 5-point Likert Scale to include the mid-point anchor point of ‘Unsure’ as graduate nurses indicated at baseline that they were unsure about some of the items on the PES-AUS tool related to their practice environment. Therefore, the aggregated score of the modified scale used in this study is not comparable to the aggregate score reported in the study by Middleton et al. [21]. Finally, self-report measures are prone to social desirability bias and this may have influenced these findings [32]. Notwithstanding these limitations, participation rates were high, with more than three-quarters of the sample completing the follow-up survey and the use of a mixed methods design enabled a more complete understanding of the experiences of new graduate nurses during their transitional support program.

## Conclusion

Ensuring the successful transition of new graduate nurses in busy acute care settings is critical to ensure a safe and competent workforce. This study has shown that while transitional support programs are helpful in supporting new graduate nurses in their first year of practice, qualitative data suggests there are still unmet needs for clinical, social and emotional support. In particular, the level of concern expressed by participants about appropriate workload and skill-mix at both baseline and follow-up suggest future research should focus on interventions to ensure effective skill-mix to better support new graduates. Understanding new graduate nurses’ experiences and their unmet needs during their first year of practice will enable nurse managers, educators and nurses to better support new graduate nurses and promote confidence and competence to practice within their scope.

## Abbreviations

AINs: Assistants in Nursing
CNEs: Clinical nurse educators
MCSS-26: Manchester Clinical Supervision Scale
MET: Medical Emergency Team
NGNs: Newly graduated registered nurses
NUMs: Nurse unit managers
PES-AUS: Practice Environment Scale – Australia
RN: Registered Nurse
SPSS: The Statistical Package for the Social Sciences
TSP: Transitional Support Program
